# Improving disease incidence estimates in primary care surveillance systems

**DOI:** 10.1186/s12963-014-0019-8

**Published:** 2014-07-26

**Authors:** Cécile Souty, Clément Turbelin, Thierry Blanchon, Thomas Hanslik, Yann Le Strat, Pierre-Yves Boëlle

**Affiliations:** 1 INSERM, UMR_S 1136, Institut Pierre Louis d’Epidémiologie et de Santé Publique, Paris F-75012, France; 2 Sorbonne Universités, UPMC Univ Paris 06, UMR_S 1136, Institut Pierre Louis d’Epidémiologie et de Santé Publique, Paris F-75012, France; 3 AP-HP, Hôpital Ambroise Paré, service de médecine interne, Boulogne-Billancourt F-92100, France; 4 Université Versailles Saint-Quentin-en-Yvelines, Versailles F-78000, France; 5 Département des maladies infectieuses, Institut de Veille Sanitaire (InVS), St Maurice F-94415, France; 6 AP-HP, Hôpital Saint-Antoine, unité de santé publique, Paris F-75012, France

**Keywords:** Surveillance, General practitioners, Sentinel network, Incidence estimation, Adjustment, Volume of consultations

## Abstract

**Background:**

In primary care surveillance systems based on voluntary participation, biased results may arise from the lack of representativeness of the monitored population and uncertainty regarding the population denominator, especially in health systems where patient registration is not required.

**Methods:**

Based on the observation of a positive association between number of cases reported and number of consultations by the participating general practitioners (GPs), we define several weighted incidence estimators using external information on consultation volume in GPs. These estimators are applied to data reported in a French primary care surveillance system based on voluntary GPs (the *Sentinelles* network) for comparison.

**Results:**

Depending on hypotheses for weight computations, relative changes in weekly national-level incidence estimates up to 3% for influenza, 6% for diarrhea, and 11% for varicella were observed. The use of consultation-weighted estimates led to bias reduction in the estimates. At the regional level (NUTS2 level - Nomenclature of Statistical Territorial Units Level 2), relative changes were even larger between incidence estimates, with changes between -40% and +55%. Using bias-reduced weights decreased variation in incidence between regions and increased spatial autocorrelation.

**Conclusions:**

Post-stratification using external administrative data may improve incidence estimates in surveillance systems based on voluntary participation.

## Background

Public health surveillance systems are nowadays expected to provide health situation awareness to officials and the population with ever increasing accuracy [1]. This is made more difficult when the population covered by the surveillance system is imperfectly characterized and potentially not representative of the general population [2],[3]. Here we investigated an approach to improve estimates using providers’ volume of activity as an external reference.

Primary care-based surveillance networks for common acute conditions are present in many countries, for example the United States Influenza-like Illness Surveillance Program (ILINet) and the European Influenza Surveillance Network (EISN) in 29 European countries. Contrary to notifiable diseases systems, which aim at exhaustive notification and may accept input from all actors in a health system, these systems are most often based on a (self-) selected sample of data providers. Yet, although the principles of functioning of such networks are essentially the same, it is striking that the results are reported somewhat differently: most countries in EISN report influenza-like illness (ILI) incidence per 100,000 inhabitants, but ILINet and some European countries report the percentage of consultations for ILI among all consultations [4]. The reason for such differences is often poorly documented. It is likely due to the long-standing issue of characterizing the population monitored by a public health surveillance system [2]. Indeed, uncertainties may exist regarding the size of the monitored population and lead to arbitrary choices in rate denominators, representativeness (i.e., the comparability of the monitored population to the general population), and finally the completeness of reporting. All these uncertainties eventually lead to bias in the reported estimates.

Theoretically, unbiased incidence estimates arise from monitoring a random sample of the general population [5],[6]. In actual surveillance networks, the monitored population may depart from a random sample because it is recruited through self-selected general practitioners (GP) or hospitals [7],[8] when they consult. Reporting raw outcomes such as the percentage of consultations with ILI allows these issues to be overlooked but fails to provide quantitative information on incidence. Providing incidence estimates requires addressing the choice of a denominator and representativeness. For example, in health systems in which patient registration is required, a denominator may be identified for each provider [2],[3],[9],[10]. Representativeness can be examined by looking for systematic variation between the covered population and the general population, or less informatively by comparing data providers to those who do not report data [5],[11]. Finally, estimates may be adjusted for underreporting using the proportion of cases consulting with a physician to obtain unbiased incidence estimates. Once these issues are resolved, estimates may be reported as time series or, more informatively, as maps [12].

Reducing bias requires making observed information as close as possible to that obtained from a random sample. This may result in weighting the original observations: more weight may be given to observations in the young if it is known that the monitored population is older than the general population. The necessary corrections are likely to depend on the disease and surveillance system, including age distributions and place of residence, among others. In this article, we propose an approach to improve incidence estimates in a voluntary GP-based surveillance network. This method is built on comparisons between volume of activity among participants in the network and others. We quantified how incidence estimates changed according to different weighting schemes at the national and regional levels.

## Methods

### The *Sentinelles* network data

The French general practitioners *Sentinelles* network is a real-time epidemiologic surveillance system based on approximately 2% of all French GPs [13]. Sentinel general practitioners (SGPs) participate to reporting on a voluntary basis. They report and describe cases of eight acute health conditions in their practice population, such as ILI, acute diarrhea (AD), or varicella (chickenpox), using a web interface or dedicated software [14],[15]. Raw reported data for years 2009 to 2011 were obtained from the *Sentinelles* network for ILI, AD, and varicella. For ILI and AD, we used the definition of epidemic period from the *Sentinelles* network [16] and used the academic year (from August to July) for varicella [17].

### National health insurance data

In France, more than 98% of the population is affiliated with the national health insurance system. Patients may freely choose their GP, but all consultations are reported to the national health insurance system for reimbursement purposes. We obtained this nearly exhaustive data on volume of consultations for all practicing French GPs and separately for SGPs, for all weeks from 2009 week 32 to 2011 week 30, in each French region (NUTS2 level: Nomenclature of Statistical Territorial Units Level 2 - including 22 regions in France, excluding overseas territories) and for six patient age groups (0-4, 5-14, 15-24, 25-44, 45-64, over 65) from the national health insurance system (CNAMTS) [18] for the entire population.

### Statistical methods

#### Comparing characteristics of SGPs and GPs

Chi-squared and Student’s t-tests were used to compare the characteristics of SGPs to that of French GPs. To investigate the relationship between physicians’ volume of consultations and number of cases reported to the system, the correlation between the average cumulated number of reported cases by SGPs during epidemic periods by region and the average number of consultations by the same SGPs over the corresponding weeks was tested using Pearson’s correlation coefficient test. The correlation was also computed by age group.

#### Horvitz-Thompson estimators

The Horvitz-Thompson estimator is often used in survey analysis to reduce bias [19]. In summary, each observation *y* is given a weight inversely proportional to its inclusion probability in the sample π. An unbiased estimator of the total *Y* of *y* obtained from a sample of *n* observations is ${\hat{Y}}_{\mathrm{HT}}=\sum_{1\leq i\leq n}\frac{1}{\pi_{i}}y_{i}$.

#### Estimating incidence from SGPs’ reports

In the *Sentinelles* network, the weekly national incidence of a disease like ILI is estimated from the number of unique patients with symptoms related to the disease reported by SGPs each week (cases). Denote *d*_*i*_*(a, t)* the number of cases of age *a* reported by SGP *i,* practicing in region *r(i)* during period *t* (could be a day, a week, or any time period); *nSGP(r(i), t)* the number of SGPs and *nGP(r(i), t)* the number of GPs in region *r(i)* during period *t*. Assuming that cases are uniformly spread between GPs, the incidence of the disease in region *r* in those aged *a* during period *t*, *I (r, a, t)*, is estimated by:

(1) $${\hat{I}}_{n}\left(r,a,t\right)=\sum_{i:r\left(i\right)=r}\frac{\mathrm{nGP}\left(r\left(i\right),t\right)}{\mathrm{nSGP}\left(r\left(i\right),t\right)}d_{i}\left(a,t\right)=\sum_{i:r\left(i\right)=r}\frac{1}{\pi_{n}\left(r\left(i\right),t\right)}d_{i}\left(a,t\right)$$

This is a Horvitz-Thompson estimator, where $\pi_{n}\left(r\left(i\right),t\right)=\frac{\mathrm{nSGP}\left(r\left(i\right),t\right)}{\mathrm{nGP}\left(r\left(i\right),t\right)}$ is the inclusion probability of SGP *i*, corresponding to the proportion of SGPs among GPs in region *r(i)* at time *t.*${\hat{I}}_{n}\left(r,a,t\right)$ is an unbiased estimator of *I (r, a, t)* under the hypothesis of uniform repartition of cases between GPs and no underreporting. This is the estimator currently used in the *Sentinelles* network. An estimate of incidence in the region *r* is ${\hat{I}}_{n}\left(r,t\right)=\sum_{a}{\hat{I}}_{n}\left(r,a,t\right)$, and estimate at the national level is obtained by summing over regions.

However, the number of cases seen by a GP could increase with his volume of consultations. In this case, the weight given to a SGP should include the number of consultations. Assuming that cases are uniformly spread over consultations, an unbiased incidence estimate for region *r*, age *a,* and period *t* is:

(2) $${\hat{I}}_{c}\left(r,a,t\right)=\sum_{i:r\left(i\right)=r}\frac{\mathrm{cGP}\left(r\left(i\right),a,t\right)}{\mathrm{cSGP}\left(r\left(i\right),a,t\right)}\;d_{i}\left(a,t\right)=\sum_{i:r\left(i\right)=r}\frac{1}{\pi_{c}\left(r\left(i\right),a,t\right)}\;d_{i}\left(a,t\right)$$

This is once again a Horvitz-Thompson estimator, where the inclusion probability of SGP *i* for those aged *a* during period *t, π*_*c*_*(r(i)*, *a*, *t)*, is proportional to the ratio of *cSGP(r(i), a, t),* the number of consultations for patients aged *a* by SGPs practicing in region *r(i)* at time *t* to *cGP(r(i), a, t),* the total number of consultations in patients aged *a* by GPs in region *r(i)* at time *t*. As above, regional and national estimates are obtained by summation.

The difference in the sampling weights in the two estimators presented above is summarized by the weight ratio $W\left(r\left(i\right),a,t\right)=\frac{\mathrm{cGP}\left(r\left(i\right),a,t\right)}{\mathrm{nGP}\left(r\left(i\right),t\right)}/\frac{\mathrm{cSGP}\left(r\left(i\right),a,t\right)}{\mathrm{nSGP}\left(r\left(i\right),t\right)}$, where *π*_*c*_(*r*(*i*), *a*, *t*) = *π*_*n*_(*r*(*i*), *t*) × *W*(*r*(*i*), *a*, *t*). *W(r(i), a, t)* is the ratio of the average number of consultations by GPs to that of SGPs in the same period, age, and region. It is independent of the monitored condition and only reflects activity of the participating SGPs. To investigate possible simplifications in the calculations, we considered four cases to compute the weight ratio *W* and estimate related incidence using ${\hat{I}}_{c}\left(r,a,t\right)$: (C1) a single weight ratio was computed at the national level for the whole period; (C2) a weight ratio *W(r(i))* was computed for each region for the whole period; (C3) a weight ratio *W(a)* was computed for each age group for the whole period; and finally (C4) where the weight ratio *W(r(i), t, a)* was computed for each week, age group, and region. To highlight differences between the four cases, analysis of variance was used to examine the significance of each component (region, age, and time) in the weights ratios.

We finally computed the change in estimated incidence $\left({\hat{I}}_{c}\left(t\right)-{\hat{I}}_{n}\left(t\right)\right)/{\hat{I}}_{n}\left(t\right)$ for each disease, region, time, and age group, as well as the coefficient of variation of the regional cumulated incidence estimates.

#### Spatial autocorrelation

We used Moran’s index to summarize spatial autocorrelation of regional incidence estimates [20]. In short, Moran’s index is the correlation coefficient of incidence in neighboring regions. Regions were neighbors if they shared a border. Positive values of the Moran’s index indicate spatial autocorrelation. We computed Moran’s index for all weeks. For ILI, we tested for an increase in Moran’s index during the epidemic period. We used McNemar’s test for paired data to determine if the (paired by week) Moran’s indices showed evidence of an increase between regional estimated incidences weighted by number of GPs and weighted by number of consultations.

## Results

SGPs participating in the surveillance network were similar compared to all GPs in age and practice of complementary medicine. SGPs were more often males (81% vs. 71%) and SGP density ranged between 0.4% and 1.2% depending on the region. The mean number of weekly consultations was also slightly different, with two additional consultations by SGPs than by GPs each week (94 vs. 92, p < 10^-12^). Interestingly, this difference was mostly due to consultations with children under 14 years old (Table 1). Apart from this difference, there was no evidence of a systematic temporal pattern between the activity of SGPs and GPs. The correlation in the weekly number of consultation for GPs and SGPs was very large (r = 0.97).

**Table 1:** **Characteristics of SGPs from the French** ***Sentinelles*** **network and all French GPs**

	**Sentinel GPs**	**French GPs**^**a**^	***p value***
Total number of GPs	442	61315	
General practitioner characteristics			
Location (n)			*< 1.10*^*-5*^
Ile-de-France	11.8% (56)	16.4%	
Northeast	17.0% (75)	18.6%	
Northwest	15.6% (69)	22.5%	
Southeast	47.0% (208)	27.2%	
Southwest	8.6% (38)	15.3%	
% Female (n)	19% (86)	29%	*< 1.10*^*-5*^
Age (mean+/-sd)	51.4 (+/-8.8)	52	*0.16*
% Complementary medicine (n/N) ^b^	13% (19/145)	12.5%	*0.7*
GP practice characteristics			
Consultations per week and age (mean+/-sd)	94 (+/- 0,3)	92	*< 1.10*^*-12*^
< 5	8	7	
5-14	9	8	
15-24	7	7	
25-44	20	20	
45-64	25	25	
≥ 65	25	25	

^a^data from CNAMTS.

^b^Missing data.

### Volume of consultations and case reports by SGPs

**Figure 1:**
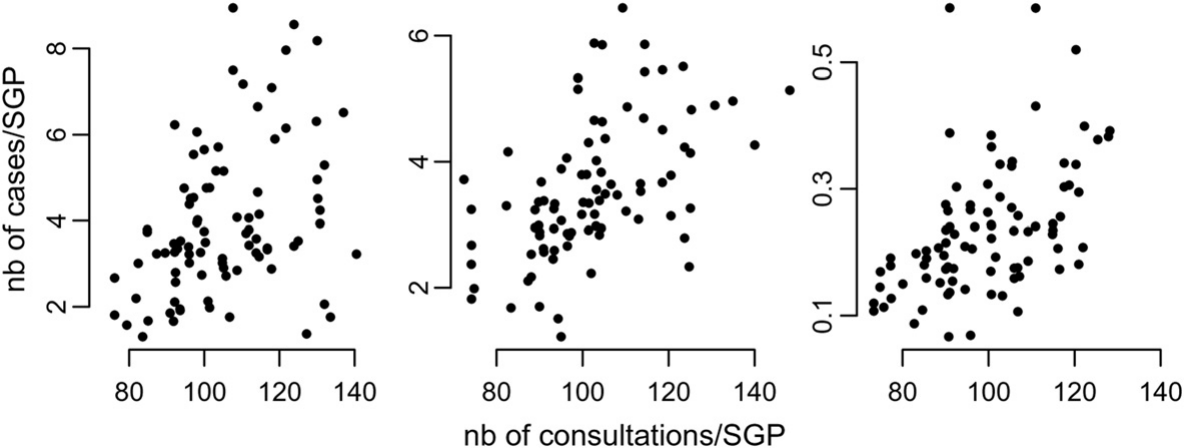
Average number of cases reported versus average number of consultations per SGP and per week for ILI (left), AD (middle), and varicella (right) in French regions.

For the three diseases, ILI, AD, and varicella, we found that a larger number of reported cases in a region was associated with a larger number of consultations in the same region, with correlation coefficients of 0.4 for ILI and 0.5 for AD and varicella (Figure 1; p < 0.001 correlation for each condition). This correlation was also found in each age group for ILI and AD and for school-aged children for varicella. This analysis is therefore supportive of using the number of consultations rather than the number of SGPs to weight contributions to incidence.

### Volume of consultation-based sampling weights

**Figure 2:**
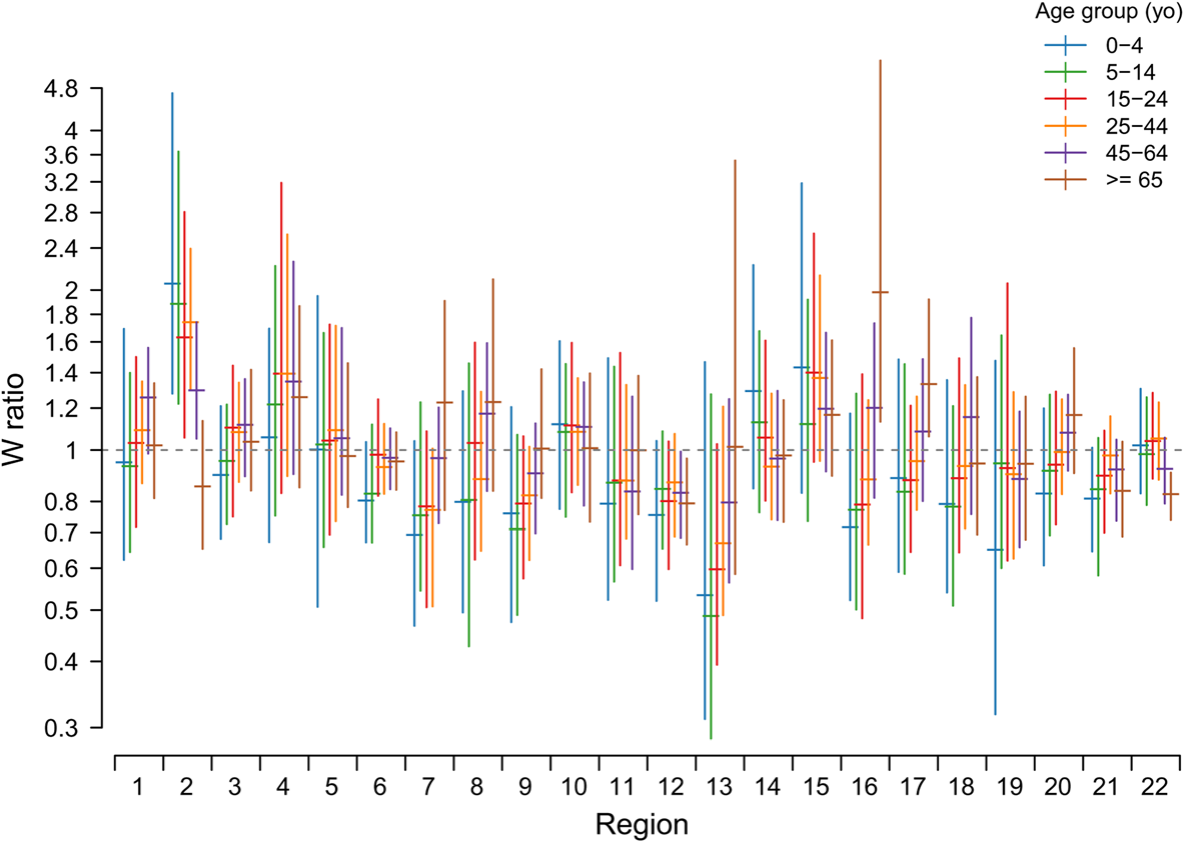
**Relative number of consultations per GP compared to SGP (weight ratio*W*) according to region, week, and age group.** Each bar shows the mean 2.5 and 97.5 quantiles of the distribution of weekly weight ratios over the two-year period (2009 week 32 to 2011 week 30).

Overall, in case C1, the simple weight ratio was 0.978 (95% PI [0.973; 0.982]), as expected by the larger number of consultations by SGPs (Table 1). The C2 weight ratios changed between regions, ranging from 0.72 to 1.23 in the 22 French regions. The weight ratios computed according to age (C3) also mirrored the differences reported in Table 1, i.e., smaller values in the young and old and values closer to 1 in adults (ranging between 0.88 and 1.02). For the C4 weight ratios computed for each week, region, and age group, approximately 95% of the values were between 0.5 and 2.0, showing that the average volume of consultations of SGPs could change from half to double that of the other practitioners, depending on region, time, and age group considered (Figure 2). The analysis of variance of C4 weight ratios highlighted the significant heterogeneity according to age group and region, as well as interactions between regions and age groups. Therefore, none of the simplifications of the weight ratio (C1, C2, C3) could properly summarize all of the differences between consultation levels in SGPs and GPs.

### Incidence estimation

**Figure 3:**
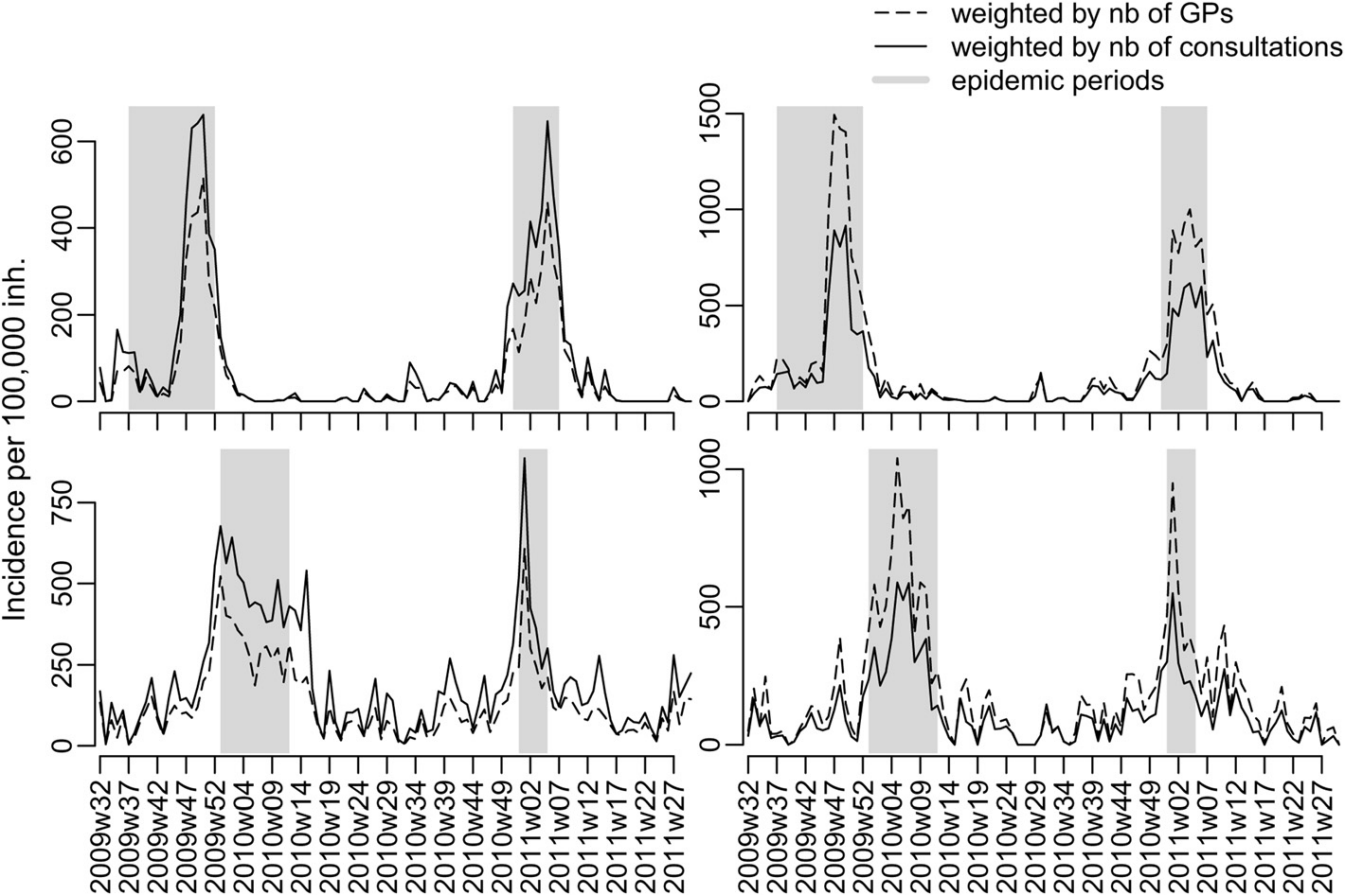
**Estimated regional incidence of GP consultations for ILI (top) and AD (bottom) using number of GPs (**${\hat{I}}_{n}\left(t\right)$**, dashed line) or number of consultations (**${\hat{I}}_{c}\left(t\right)$**, normal line) in the two regions with the most extreme changes.**

**Figure 4:**
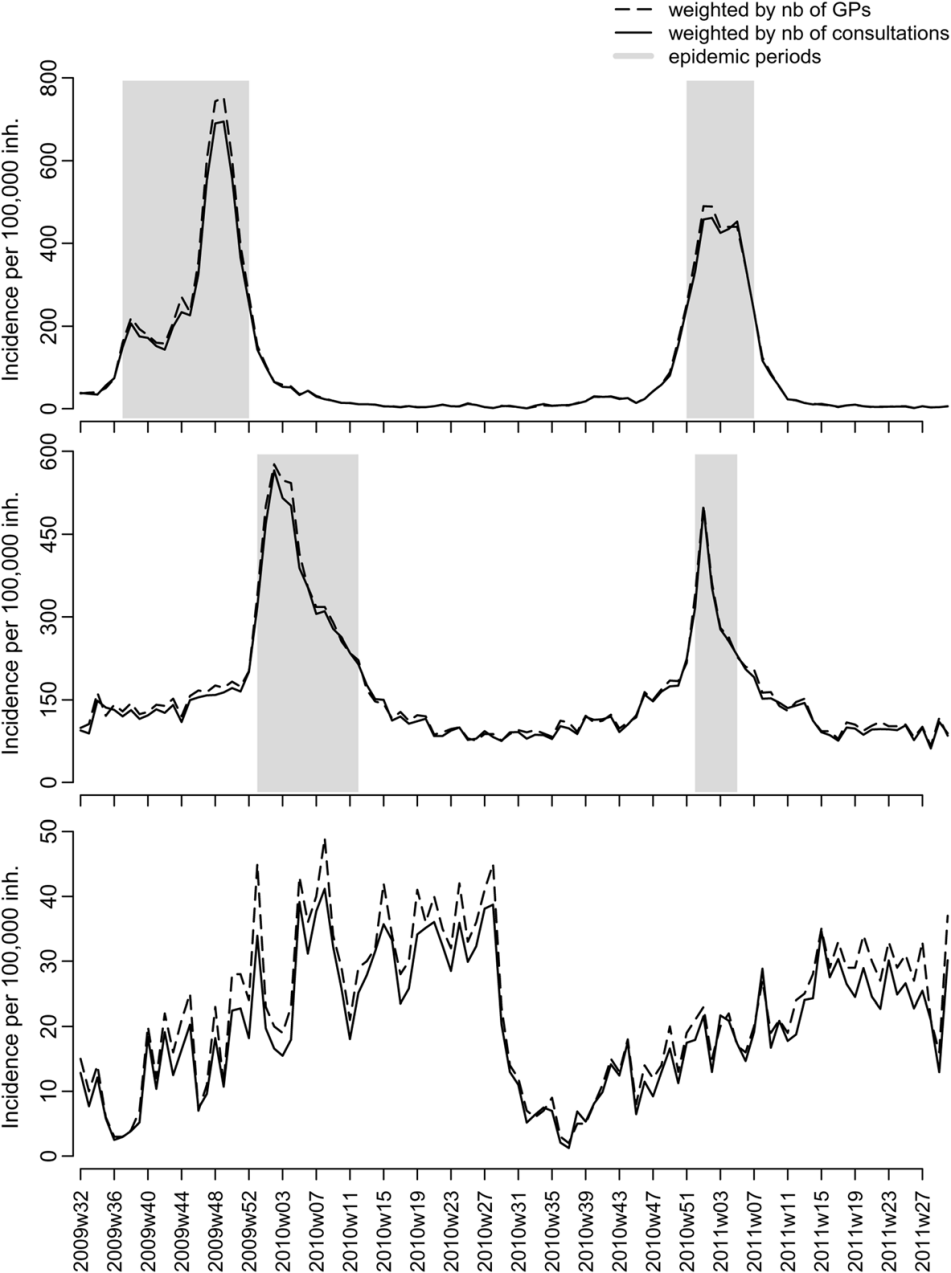
**Estimated French national incidence of GP consultations for ILI (top), AD (middle), and varicella (bottom) using number of GPs (**${\hat{I}}_{n}\left(t\right)$**, dashed line) or number of consultations (**${\hat{I}}_{c}\left(t\right)$**, normal line).**

Incidence estimates showed a large variability at the regional level according to the choice of the weighting scheme. The most extreme differences between consultation-weighted and GP-weighted regional incidence estimates were a reduction by 35% for ILI and 40% for AD and an increase up to 54% for ILI and 55% for AD when using weights based on region, age group, and time (Figure 3). As a brief summary of the scenarios for weight computations, we report the differences in the national-level incidence estimates for the three diseases: the simple overall weight (C1) led to a relative reduction of 2% of all disease incidence estimates compared to adjustment with the number of GPs only; the reduction was larger when weights were computed at the regional level (C2), with reduction by 3.5% (AD), 3% (ILI), and 4% (varicella); and it was somewhat larger using weights summarizing differences in the age of patients (C3): 4% (AD), 5% (ILI), and 11% (varicella). Finally, using age and region differences (C4), the decrease was 3% for AD, 6% for ILI, and 11% for varicella (Figure 4). For ILI and AD, the largest absolute differences between estimates were found at the epidemic peaks, corresponding to a decrease of 60 cases per 100,000 inhabitants for ILI (from 760 to 700) and 40 cases per 100,000 inhabitants for AD (from 540 to 500). Differences were somewhat smaller for varicella (about seven fewer cases per 100,000 inhabitants). The relative decrease was the same over the seasons, with larger variability for ILI during spring and summer weeks.

Overall, the coefficient of variation of regional estimates was the largest using estimates with adjustment on the number of GPs. Depending on the year and disease, it ranged from 26% to 48% for cumulated incidence estimates, showing that large regional variability was present in the reported incidences. Using estimates adjusted on the number of consultations led to a reduction in the coefficient of variation, i.e., made incidence more commensurate between regions. Using weight C4, the largest coefficient of variation was reduced from 48% to 39%, showing that part of the between-regions variability disappeared as the characteristics of the GPs were taken into account. The reductions in coefficient of variation were the largest for influenza and varicella and were more limited for diarrhea.

### Spatial autocorrelation

**Figure 5:**
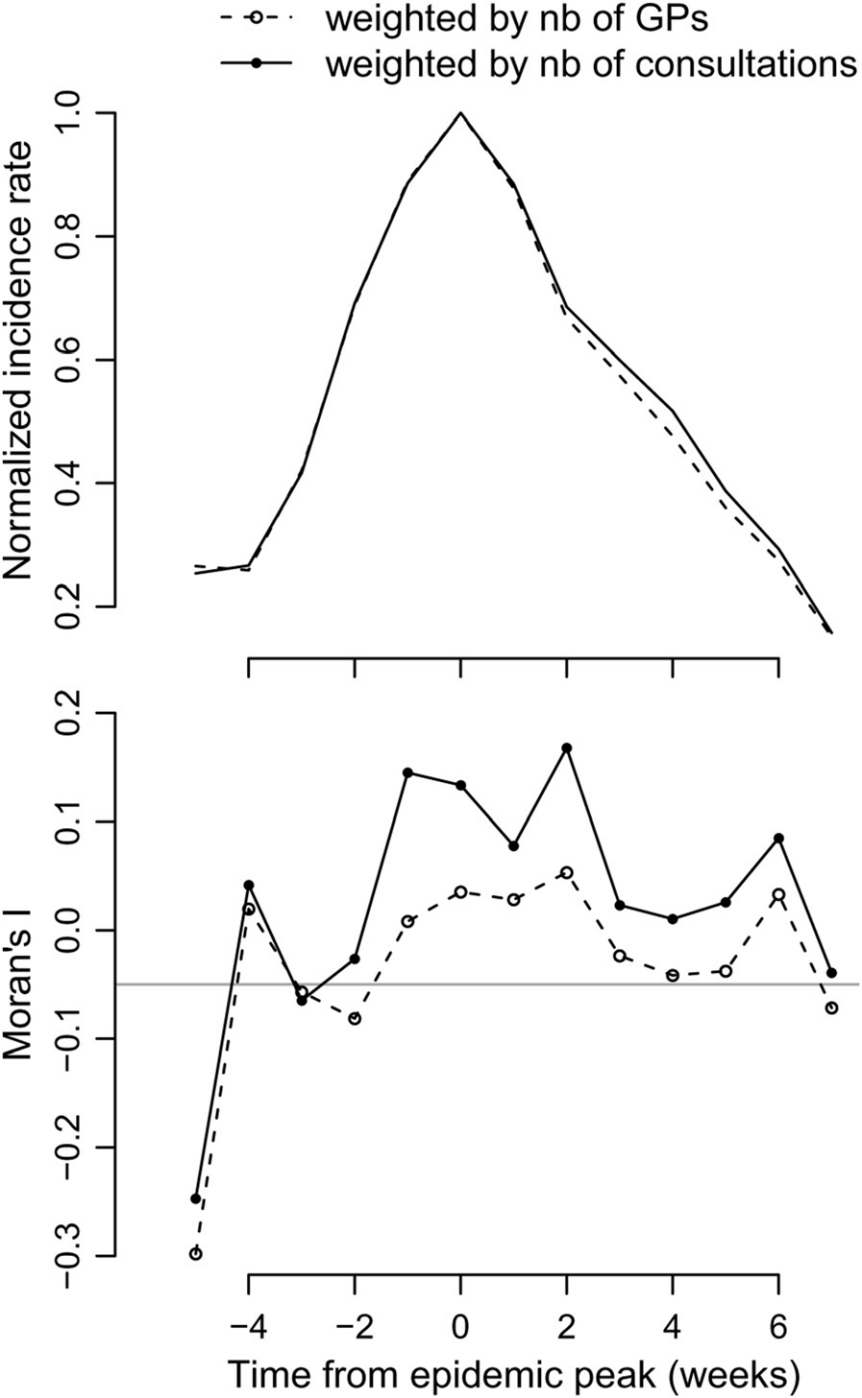
**Autocorrelation in regional ILI incidence during an epidemic period.** (top) National ILI epidemic profile. Week 0 is defined as the epidemic peak. (bottom) Moran’s index computed from regional incidence post-stratified on the number of GPs (${\hat{I}}_{n}\left(r,t\right)$, dashed) or on the number of consultations (${\hat{I}}_{c}\left(r,t\right)$, plain). The horizontal grey line shows the expected value without spatial autocorrelation.

There was evidence of spatial autocorrelation in incidence for ILI but not for AD and varicella. More precisely, Moran’s index was larger than expected by chance for a couple of weeks during ILI epidemics (Figure 5). Importantly, we found that Moran’s index for ILI increased using regional estimates based on weight C4 compared to those based on number of GPs (p < 0.001), indicating larger spatial autocorrelation in these estimates.

## Discussion

Reducing bias in surveillance systems is a first step for improving public health decisions. Here, we have highlighted that post-stratification using external data improves incidence estimates for acute diseases like ILI, AD, and varicella. These bias reduced estimates may be used to provide improved spatial and national information.

Although the major issue of representativeness for surveillance systems is to compare the monitored population to the general population, comparisons are often limited to that of participating GPs to others [10]. Here, in the French *Sentinelles* network, participating SGPs were similar to other GPs in a number of ways (age, practice of complementary medicine), but differed in some respects: they were more frequently males, were not equally spread over the territory, and they saw more patients each week. Self-selection of data providers participating in the surveillance system can lead to such differences, either by chance alone or because participation depends on providers’ characteristics. Bias should only incur if the probability of a GP reporting a case of disease is related to these characteristics. For example, having more male SGPs could be an issue, as some conditions are more likely to be reported to a female GP than to a male GP [21]. The conditions monitored here are unlikely candidates for such differential reporting, making this sex imbalance irrelevant for population representativeness. French female GPs also more often work part-time than male GPs [22], but as this directly leads to variation in the number of consultations, incidence estimates weighted using the volume of consultations must correct for this imbalance. A last issue is that participation in a research or surveillance network could lead to systematic differences between participating GPs and others. But, if different patterns of prescription have been reported between GPs participating in research and others [23],[24], fewer differences concerned the case-mix of patients. For example, the prevalence of 11 common chronic diseases was almost the same in GPs who were taking part in surveillance and those who were not [25]. We assumed that this would be the case for common acute conditions like ILI, AD, or varicella, especially as a detailed case definition was used. A systematic identification of all characteristics that would lead to differences in reporting between SGPs and GPs is difficult in practice, as information on potential (but non-participating) providers is seldom available. As differences in the volume of consultations explained approximately 20% of the variance in cases reports, other characteristics of interest may exist. A final caveat is that repeat consultations with GPs by the same patient for the same disease episode could bias incidence estimates. However, SGPs would only report patients once per episode, and consultations with several GPs by the same patient is rare in the French system as it leads to lower reimbursements by the social security system.

Apart from systematic differences due to the characteristics of the participating GPs, a further problem in computing incidence is the lack of a proper denominator. In health systems based on registration of patients with a practice, it may be possible to use the size of the patient list [2],[10]. In the French health insurance system, free choice of the GP and absence of registration makes this approach infeasible. However, more than 99% of the 60,000 GPs participate in the national health insurance system. This very large coverage means that administrative data on reimbursements for consultations charged to patients provide a very good picture of the activity of all French GPs. Moreover, it makes the whole population a sensible denominator in the end, as very little primary care medical activity is excluded from the national health insurance system data.

Improving estimates for surveillance networks, especially to provide better spatial estimates, may resort to different solutions. The first is “by design”, choosing data providers to maximize coverage and representativeness, using methods of operational research and panel design [6],[26]-[28]. These approaches are difficult to apply when participants are voluntary GPs, whose reasons to participate and survival in the system are poorly characterized [29]. In this case, estimates may be improved by introducing weights in the computations to mimic a sample of the general population [11], leading to “post-stratified” Horvitz-Thomson estimators. Identifying and collecting relevant external reference to compute weights is a first required step. In most countries, the readily available administrative reimbursement data may provide such reference. A limitation is that direct assessment of the amount of bias reduction is seldom feasible as the targeted value remains unknown in practice. Poorly constructed post-stratified estimates should not increase bias, but they may be more noisy [30]. Here, the correlation observed between volume of consultations and number of cases in SGPs clearly supported the choice of using volume of consultations for post-stratification. Additional support for these weights comes from the reduction of the coefficient of variation of regional estimates of disease incidence, a desirable feature as the cumulated incidence of varicella between French regions, for example, is not expected in the absence of universal vaccination. The increased spatial autocorrelation found with ILI incidence is also relevant, as commuting makes epidemics in nearby regions more similar [31]. Subject to data availability, we defined post-stratification weights at the NUTS2 level and with six age classes. Using tools of spatial analysis could help select the best scale of definition [32].

As adjustment on the number of participating GPs has been the practice in the *Sentinelles* network, comparisons of the consultations based estimates are warranted. For ILI and AD, the largest absolute impact on incidence estimates was found near the peak of incidence, although the relative impact was more constant throughout the year. Interestingly, the extent of the changes between the various estimators was not major at the national level and would not have changed the detection of periods of epidemic circulation over the years considered. For varicella, the difference in age of patients in SGPs and GPs had a larger impact. We found that age-based weights (C3) led to almost the same estimates as with C4 at the national level; this is noteworthy given weights in C3 can be calculated once and used over again, while those in C4 must be updated in real time every week. Finally, regional estimates based on the C4 scheme would improve the quality of the maps based on kriging incidence [12], as input data would be bias-reduced. This lastly highlights that defining an epidemic or alert threshold applicable at all scales will be dependent on how weights are defined. Here, the reduced variability of regional incidence estimates and increased spatial auto-correlation using sampling weights based on volume of consultations stratified on time, age, and regional information (C4) makes standardization of epidemic threshold definitions more likely.

## Conclusions

We have described a method to improve estimators for the incidence of acute conditions in provider-based public health surveillance systems. Our study suggests that administrative data regarding activity of data providers may have a strong impact. This makes unbiased measurement of population health possible at refined spatial resolution and can strengthen confidence and usefulness in results from population-based surveillance systems.

### Consents

“Data collection and treatment conformed with French regulations (authorization from the French Data Protection Agency number #471393)”.

## Abbreviations

GP: General practitioners

SGP: Sentinel general practitioners

ILI: Influenza-like illness

AD: Acute diarrhea

## Competing interests

The authors declare that they have no competing interests.

## Authors’ contributions

CS, PYB, YLS, and CT designed methods. CS performed analysis. CS and PYB wrote the manuscript. CT, YLS, TB, and TH revised the manuscript. All authors read and approved the final manuscript.
